# Feasibility Study of a Network Meta-Analysis and Unanchored Population-Adjusted Indirect Treatment Comparison of Niraparib, Olaparib, and Bevacizumab as Maintenance Therapies in Patients with Newly Diagnosed Advanced Ovarian Cancer

**DOI:** 10.3390/cancers14051285

**Published:** 2022-03-02

**Authors:** Domenica Lorusso, Holly Guy, Yevgeniy Samyshkin, Carol Hawkes, Kasey Estenson, Robert L. Coleman

**Affiliations:** 1 Fondazione Policlinico Gemelli of Rome, 00168 Rome, Italy; 2 Department of Gynecologic Oncology, Catholic University of Sacred Heart, 00168 Rome, Italy; 3 FIECON Ltd., St Albans AL3 4PA, UK; holly.guy@fiecon.com; 4 GlaxoSmithKline, Brentford TW8 9GS, UK; yevgeniy.x.samyshkin@gsk.com (Y.S.); carol.l.hawkes@gsk.com (C.H.); 5 GlaxoSmithKline, Philadelphia, PA 19112, USA; kasey.x.estenson@gsk.com; 6 Eisai Inc., Nutley, NJ 07677, USA; 7 Texas Oncology, US Oncology Research, The Woodlands, TX 77380, USA; robert.coleman@mckesson.com

**Keywords:** ovarian cancer, first-line maintenance therapy, niraparib, bevacizumab, NMA, olaparib

## Abstract

**Simple Summary:**

Ovarian cancer (OC) is a leading cause of cancer-related death and 85% of women with advanced OC relapse after chemotherapy. First-line (1L) maintenance therapy is given to prolong the benefit of chemotherapy. However, selection of a 1L maintenance therapy is challenging given the number of therapies available and the lack of clinical trials that directly compare these therapies. Indirect treatment comparisons (ITCs) allow the comparison of therapies across trials and may inform selection of the most appropriate treatment option. ITCs must follow statistical principles to ensure similarity among trials and allow for a fair comparison. This study assessed whether two types of ITC could be performed to compare the poly(ADP-ribose) polymerase inhibitor niraparib with other 1L maintenance therapies. The 12 clinical trials assessed differed too significantly to meet recommended criteria for comparison. This study highlights the need for caution when comparing trial data to inform treatment decisions.

**Abstract:**

Selecting a first-line (1L) maintenance option for ovarian cancer is challenging given the variety of therapies, differing trials, and the lack of head-to-head data for angiogenesis and poly(ADP-ribose) polymerase (PARP) inhibitors. Thus, indirect treatment comparisons (ITCs) can aid treatment decision making. This study assessed the feasibility of two ITCs, a network meta-analysis (NMA) and a population-adjusted ITC (PAIC), comparing the efficacy of the PARP inhibitor niraparib in the PRIMA trial (NCT02655016) with other 1L maintenance treatments. A systematic literature review was conducted to identify trials using the Cochrane Handbook for Systematic Reviews of Interventions to assess differences in trial design, population characteristics, treatment arms, and outcome measures. All 12 trials identified were excluded from the NMA due to the absence of a common comparator and differences in survival measures and/or inclusion criteria. The PAIC comparing PRIMA and PAOLA-1 trials was also not feasible due to differences in inclusion criteria, survival measures, and the previous receipt of chemotherapy/bevacizumab. Neither ITC met recommended guidelines for analysis; the results of such comparisons would not be considered appropriate evidence when selecting 1L maintenance options in ovarian cancer. ITCs in this setting should be performed cautiously, as many factors can preclude objective trial comparisons.

## 1. Introduction

In the age of precision medicine, oncologists have a variety of therapeutic options, supported by a large amount of clinical data, and are challenged to select the optimal therapy based on the benefit:risk profile for each patient, while also considering the uncertainty of their disease course [1]. For oncologists who treat women with ovarian cancer, this is a particular challenge when selecting a maintenance therapy following first-line (1L) chemotherapy [2]. Ovarian cancer is a leading cause of cancer death in women [3], even though advances in treatment options have led to improved outcomes in women with advanced epithelial ovarian cancer [4,5,6]. However, because up to 85% of women with advanced ovarian cancer relapse after standard 1L chemotherapy, there remains a high unmet need to achieve disease control and lasting remission after 1L treatment [7,8].

Several randomized controlled trials (RCTs) have demonstrated the benefit of 1L maintenance therapy in delaying disease recurrence or progression and prolonging the time between chemotherapy regimens, which is an important predictor of response to subsequent treatments [3,7,9,10]. Maintenance therapy following 1L and/or recurrent treatment is endorsed by ovarian cancer treatment guidelines developed by the European Society for Medical Oncology, American Society of Clinical Oncology, and National Comprehensive Cancer Network [11,12,13,14]. However, there is no single consensus algorithm, and these treatment pathways vary widely in their recommendations for selecting specific maintenance therapy options [11,12,13,14]. Poly(ADP-ribose) polymerase (PARP) inhibitors, including niraparib, rucaparib, and olaparib, have revolutionized the treatment of advanced ovarian cancer [3]. These agents provide maintenance therapy options that prolong progression-free survival (PFS), have manageable toxicity profiles, and delay the subsequent use of chemotherapy and the impact of the associated toxicities on quality of life in women with advanced ovarian cancer [3,7,9,15].

Niraparib monotherapy has shown clinical benefit as a 1L maintenance therapy in PRIMA (NCT02655016) and is approved in the US and EU regardless of biomarker status [16,17,18]. In the PAOLA-1 trial (NCT02477644), the combination of olaparib and bevacizumab (anti-angiogenic therapy) demonstrated a clinical benefit in the 1L maintenance setting and is approved for patients with homologous recombination-deficient (HRd) ovarian cancer [19,20,21]. Bevacizumab can be given with chemotherapy in the 1L setting, with treatment continuing into the maintenance phase [9]. The RCTs for available 1L maintenance treatment were designed to address different unmet needs in ovarian cancer and therefore differ considerably in their study populations and designs [4]. A standardized, objective method is needed to inform a relative comparison of 1L maintenance therapy options when data cannot be readily compared across studies [3].

**Figure 1:**
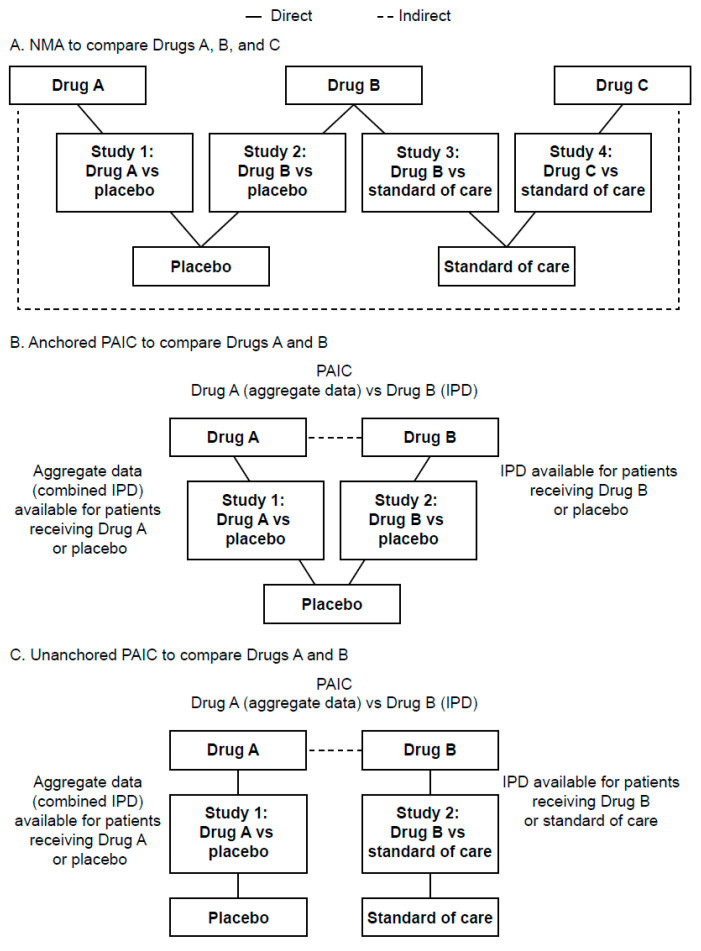
Example schematics of NMA and anchored or unanchored PAICs. Example schematic of (**A**) NMA to compare Drugs A, B, and C, (**B**) anchored PAIC to compare Drugs A and B, and (**C**) unanchored PAIC to compare Drugs A and B. —indicates direct comparison; —indicates indirect comparison. IPD, individual patient data; NMA, network meta-analysis; PAIC, population-adjusted indirect treatment comparison.

In the absence of head-to-head trials that directly compare treatments, indirect treatment comparisons (ITCs) may be used to inform the relative efficacy of therapies evaluated in separate trials and raise new hypotheses to be tested [22]. To conduct an ITC, the available evidence can be mapped out in a meta-analysis [22]. If more than two clinical trials are being compared that include multiple therapies and comparators, a network meta-analysis (NMA) is used to show the multiple pair-wise (i.e., studies with the same comparator arm) comparisons across different therapies (Figure 1A) [22]. NMAs require that the evidence from RCTs forms a connected network for the outcome of interest (Figure 1A) [22,23]. NMAs are based on a strict similarity assumption, which dictates that the RCTs must be similar in terms of design, population, interventions, outcomes of interest, and treatment effect modifiers [22,23]. Treatment effect modifiers (also referred to as predictive factors) are variables that can influence the outcome of a particular therapy [22,23,24]. These differ from prognostic factors, which can reflect the general outcome of a cohort irrespective of specific treatment [24,25]. In ovarian cancer, *BRCA* mutation (*BRCA*m) serves both classifiers, as it is a prognostic factor for improved survival outcomes and a predictive factor for enhanced response to PARP inhibitors in the maintenance setting [26].

Some alternatives to NMAs are population-adjusted indirect comparisons (PAICs), which can be used when there is an imbalance of treatment effect modifiers between RCTs [24]. PAICs use individual patient data (IPD) for at least one of the trials to adjust for imbalances in treatment effect modifiers and minimize bias in outcomes [24]. PAICs can be described as anchored (trials share a common comparator) or unanchored (trials have different comparators) (Figure 1B,C) [24].

The use of meta-analyses in oncology is increasing but interpretation of the data can be misleading if these analyses are not conducted properly [27]. In this study, we assessed whether an NMA was feasible to estimate the comparative efficacy of niraparib in PRIMA versus other 1L maintenance RCTs in patients with advanced ovarian cancer. The feasibility of a PAIC comparing PRIMA with the PAOLA-1 study of olaparib plus bevacizumab was also evaluated. The potential implications of this study span across the field of oncology, as they may help educate oncologists about important considerations when comparing RCT data to inform their treatment decisions.

## 2. Materials and Methods

The trials included in the NMA and PAIC analyses were based on a systematic literature review (SLR) conducted in February 2020 to identify RCTs evaluating maintenance therapy in patients with ovarian cancer who had received only one line of previous chemotherapy. The SLR search terms are listed in Supplementary Tables S1–S3. Trials evaluating chemotherapeutic agents given as maintenance therapy, such as paclitaxel, paclitaxel poliglumex, topotecan, or hexamethylmelamine, were excluded because the intent was to evaluate maintenance therapy following active chemotherapy treatment and not continued chemotherapy [28,29,30,31,32]. Additional details on the SLR methodology and outcomes are reported separately [33].

Guidelines from the Cochrane Handbook for Systematic Reviews of Interventions [23] were used to assess the feasibility of an NMA based on the level of heterogeneity across RCTs by comparing study designs, population characteristics, treatment arms, and outcome measures. Specific factors that might result in heterogeneity are outlined in Table 1.

The feasibility of an unanchored PAIC for PRIMA (using IPD) and PAOLA-1 (using published aggregate data [19,34]) was assessed based on the key assumptions outlined in the guidance by the Decision Support Unit (DSU) in the National Institute for Health and Care Excellence (NICE) DSU Technical Support Document 18 [35]. Violations of these assumptions result in biased or spurious estimates. In addition to the assumptions required for standard NMAs, unanchored PAICs also require conditional constancy of absolute effects, which means that all treatment effect modifiers and prognostic factors for the trials being compared are known and do not change throughout the trials [24]. Identification of these factors and their availability in the trials was therefore the key consideration of the feasibility assessment. This analysis considered the feasibility of an NMA to compare PFS and overall survival (OS) outcomes and a PAIC to compare PFS outcomes.

The presence of visible residual disease (VRD, based on history of cytoreductive surgery) was considered a key treatment effect modifier and prognostic factor in these analyses that would influence efficacy outcomes [16,36]. Additional treatment effect modifiers or prognostic factors considered included: age (mean), tumor histology (% serous histology), Eastern Cooperative Oncology Group performance status (% status 0), International Federation of Gynecology and Obstetrics (FIGO) stage (% stage II or stage IV), history of cytoreductive surgery, best response to most recent platinum-based chemotherapy (% partial response), *BRCA*m status (% positive), HRd status (% positive), prior treatment exposure alongside chemotherapy (% received bevacizumab), receipt of neoadjuvant chemotherapy (NACT; % receiving), and cancer antigen-125 (CA-125) levels ≤ the upper limit of normal (%).

## 3. Results

### 3.1. NMA Feasibility Assessment

**Figure 2:**
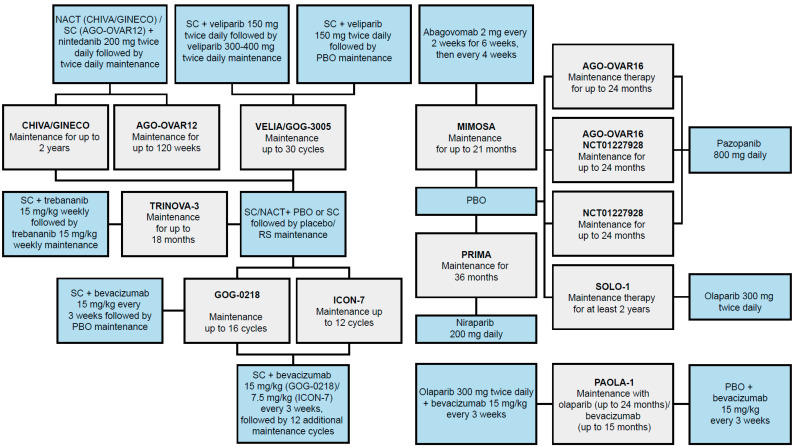
Full potential network of identified RCTs for NMA feasibility [33]. Blue boxes indicate treatment regimens assessed in studies identified in the NMA feasibility analysis. Gray boxes represent RCTs (trial name in bold); studies may have had multiple treatment arms, indicated by multiple branches to blue treatment boxes. The treatment duration of each study is listed in gray boxes. NACT, neoadjuvant chemotherapy; NMA, network meta-analysis; PBO, placebo; RCT, randomized controlled trial; RS, routine surveillance; SC, standard chemotherapy.

The SLR identified 12 RCTs, including PRIMA, that evaluated maintenance therapy following 1L chemotherapy for inclusion in the NMA [33]. The full potential network of RCTs is depicted in Figure 2. These trials included eight monotherapy options comprised of PARP inhibitors (niraparib, olaparib, and veliparib), a peptide inhibitor (trebananib), an anti-CA-125 monoclonal antibody (mAb; abagovomab), tyrosine kinase pathway inhibitors (pazopanib and nintedanib), an anti-vascular epithelial growth factor mAb (bevacizumab), and one combination regimen (olaparib plus bevacizumab). To date, only niraparib, olaparib, and bevacizumab plus olaparib have been approved for use as maintenance therapies following 1L chemotherapy [37,38,39]. Bevacizumab is also approved in combination with 1L chemotherapy and then for monotherapy maintenance [12,14].

Of the 12 RCTs identified, 6 trials evaluated maintenance therapies initiated after 1L chemotherapy, including niraparib (PRIMA [16,40,41,42,43]), olaparib (SOLO-1 [44,45,46,47,48,49,50,51,52,53,54,55,56]), abagovomab (MIMOSA [57,58]), pazopanib (AGO-OVAR16 [59,60,61,62,63,64,65] and NCT01227928 [66,67]), and olaparib added to bevacizumab after 1L chemotherapy (PAOLA-1 [19,34]). The remaining RCTs evaluated bevacizumab (ICON-7 [68,69] and GOG-0218 [70,71]), nintedanib (CHIVA/GINECO [37] and AGO-OVAR12 [72]), trebananib (TRINOVA-3 [73]), or veliparib (VELIA/GOG-3005 [74,75]) as maintenance therapies initiated with 1L chemotherapy and continuing into a maintenance phase.

Upon review, all 12 RCTs were excluded from this feasibility assessment due to heterogeneity in either the study design, patient population, and/or outcomes compared with PRIMA [16,40,41,42,43] (Table 2).

#### 3.1.1. Study Design Heterogeneity

Therapies that were evaluated as maintenance therapies initiated alongside 1L chemotherapy, followed by a maintenance phase, cannot be compared with PRIMA [16,40,41,42,43], in which niraparib was initiated following 1L chemotherapy. For therapies initiated with 1L chemotherapy, it is not possible to elucidate the contribution of the agent to the maintenance phase from that in the 1L chemotherapy phase. Patient selection differed between PRIMA [16,40,41,42,43], which required a clinical response to 1L chemotherapy, and therapies initiated with 1L chemotherapy, which did not. Therefore, ICON-7 [68,69], GOG-0218 [70,71], TRINOVA-3 [73], VELIA/GOG-3005 [74,75], CHIVA/GINECO [37], and AGO-OVAR12 [72] were excluded from an NMA with PRIMA [16,40,41,42,43].

Time on treatment can vary based on the maximum treatment duration specified in the treatment discontinuation rules. For instance, the maximum treatment duration was 24 months for olaparib in PAOLA-1 [19,34] and 36 months for niraparib in PRIMA [16,40,41,42,43]. If a large proportion of patients terminated therapy prior to disease progression, the outcome of PFS may be impacted by the shorter treatment regimen in addition to other variables discussed below. The maximum treatment durations were substantially shorter for AGO-OVAR16 [59,60,61,62,63,64,65], NCT01227928 [66,67], and TRINOVA-3 [73] compared with PRIMA [16,40,41,42,43]. ICON-7 [68,69], SOLO-1 [44,45,46,47,48,49,50,51,52,53,54,55,56], PAOLA-1 [19,34], and TRINOVA-3 [73] all reported a longer median follow-up compared with PRIMA [16,40,41,42,43]. Despite comparable treatment arms, MIMOSA [57,58] was excluded because treatment was discontinued based on recurrence (defined as the appearance of any lesion or the development of tumor-related symptoms evaluated by medical examination and confirmed by a documented CT-scan every 12 weeks) rather than disease progression (per RECIST version 1.1) as used in PRIMA [16,40,41,42,43].

#### 3.1.2. Patient Population Heterogeneity

When considering heterogeneity within the intention-to-treat patient population at baseline, all RCTs had confounding factors. VRD is a key treatment effect modifier, as patients without VRD following primary debulking surgery (PDS) have a better prognosis than patients with VRD, in particular those with stage III disease [16,36]. MIMOSA [57,58], AGO-OVAR16 [59,60,61,62,63,64,65], PAOLA-1 [19,34], SOLO-1 [44,45,46,47,48,49,50,51,52,53,54,55,56], VELIA/GOG-3005 [74,75], NCT01227928 [66,67], CHIVA/GINECO [37], and TRINOVA-3 [73] were excluded on the basis of including patients without VRD following primary debulking surgery. PRIMA [16,40,41,42,43] differed from these studies in that it excluded patients with stage III disease without VRD following primary debulking surgery. Additionally, SOLO-1 [44,45,46,47,48,49,50,51,52,53,54,55,56] only included patients with *BRCA*m, whereas PRIMA [16,40,41,42,43] included patients regardless of biomarker status. A connected NMA is not feasible if there are differences in patient populations that cause an imbalance in treatment effect modifiers, including the presence of VRD or differences in *BRCA*m status.

#### 3.1.3. Outcome Heterogeneity

Following a review of the heterogeneity of the PFS outcome across the 12 RCTs, all trials were excluded. PFS assessed by a blinded independent committee review (BICR) was the primary endpoint in PRIMA [16,40,41,42,43] but PFS was investigator-assessed in SOLO-1 [44,45,46,47,48,49,50,51,52,53,54,55,56], AGO-OVAR16 [59,60,61,62,63,64,65], and PAOLA-1 [19,34]. In previous studies, a good concordance was observed in PFS outcomes assessed by BICR compared with investigator assessment [76,77,78]. Therefore, differences in PFS assessments may not influence trial outcomes, but still serve as a potential source of study design heterogeneity that should be considered when conducting an NMA. PFS was not assessed in MIMOSA [57,58] and insufficient PFS data were reported in CHIVA/GINECO [37] and AGO-OVAR12 [72]. ICON-7 [68,69], GOG-0218 [70,71], TRINOVA-3 [73], VELIA/GOG-3005 [74,75], CHIVA/GINECO [37], and AGO-OVAR12 [72] were excluded because PFS included the time patients were receiving standard chemotherapy; as such, the PFS timings were inconsistent. OS assessments were also inconsistent with the method of assessment used for PRIMA [16,40,41,42,43] (i.e., some studies included the time period during which patients received 1L chemotherapy) or were immature at the time of this analysis and, as a result, all 12 RCTs were excluded.

### 3.2. PAIC Feasibility Assessment

**Figure 3:**
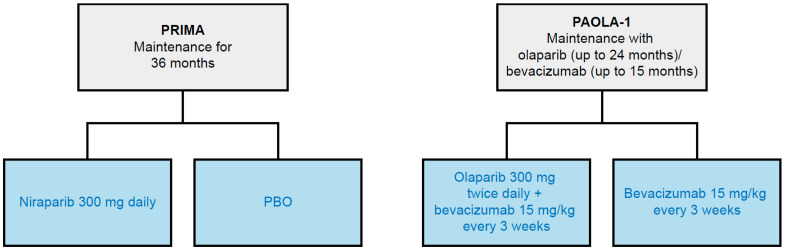
Network of identified RCTs for PAIC feasibility. Gray boxes represent RCTs (trial name in **bolded** text) included in the PAIC feasibility analysis, with treatment duration noted. Blue boxes indicate treatment regimens assessed; studies may have had multiple treatment arms, indicated by multiple branches. PAIC, population-adjusted indirect treatment comparison; PBO, placebo; RCT, randomized controlled trial.

The SLR identified the PAOLA-1 [19,34] trial of olaparib plus bevacizumab as a comparator of interest for a PAIC with the PRIMA [16,40,41,42,43] trial of niraparib. An unanchored PAIC was assessed given that PRIMA [16,40,41,42,43] and PAOLA-1 [19,34] do not share a common comparator arm (Figure 3). There was some overlap between the two studies regarding design, blinding, cross-over, and location. In both trials, patients were blinded and randomized (2:1) to either investigational treatment or control. However, it was determined that an unanchored PAIC was not feasible due to significant differences between PRIMA [16,40,41,42,43] and PAOLA-1 [19,34] in terms of trial outcomes, inclusion/exclusion criteria, the use of bevacizumab prior to the study, and the use of NACT.

#### 3.2.1. Inclusion/Exclusion Criteria

The wider inclusion criteria in PAOLA-1 [19,34] (including patient cytoreductive surgery history and best response to most recent platinum-based therapy) meant that a proportion of the PAOLA-1 population was expected to have a “better prognosis” than the PRIMA [16,40,41,42,43] population. In PAOLA-1 [19,34], patients with FIGO Stage III with or without evidence of disease after PDS were included in the study. Approximately half of the patients in PAOLA-1 [19,34] receiving olaparib plus bevacizumab (54%) or the comparator (placebo plus bevacizumab: 52%) had no evidence of disease after PDS. However, in PRIMA [16,40,41,42,43], patients with FIGO Stage III disease were only eligible if they had VRD following PDS. Therefore, patients in PRIMA [16,40,41,42,43] with FIGO Stage III and VRD had a “worse prognosis” at baseline compared with patients in PAOLA-1 [19,34] who were FIGO Stage III without evidence of disease. This lack of overlap between the trial populations violates the “conditional constancy of absolute effects” assumption, making an unanchored PAIC not feasible. Although the recently published high-risk population from PAOLA-1 [79] appears to be a more similar population to the PRIMA population [16,40,41,42,43], a number of factors preclude a robust comparison with PRIMA, including differences in the frequency of PFS assessment and the prior use of bevacizumab alongside chemotherapy, which are described below.

#### 3.2.2. Bevacizumab Treatment Prior to Study Entry

Patients in PAOLA-1 [19,34] must have had a response to prior bevacizumab in combination with platinum-based chemotherapy before study entry; patients then continued with bevacizumab as a maintenance therapy with added olaparib or placebo. Few patients in PRIMA [16,40,41,42,43] (*n* = 7) received bevacizumab prior to commencing niraparib maintenance therapy. This difference between the two studies is a potential confounding factor and source of bias and uncertainty.

#### 3.2.3. Receipt of NACT

In PRIMA [16,40,41,42,43], 66% of patients treated with niraparib in the intention-to-treat group and 67% of *BRCA*m patients received NACT [80]; however, the proportion of patients in PAOLA-1 [19,34] who received NACT was not reported. Use of NACT was identified as a potential confounding factor for this analysis; therefore, the proportion of patients who received NACT should be similar for a valid comparison of these trials [81]. Given its prognostic value and the lack of uniform reporting across both studies, a comparison between PRIMA [16,40,41,42,43] and PAOLA-1 [19,34] populations could be biased.

#### 3.2.4. PFS Method of Assessment and Frequency of Measurement

The primary endpoint for PRIMA [16,40,41,42,43] was PFS by BICR, whereas the primary endpoint for PAOLA-1 [19,34] was investigator-assessed PFS. Disparities in these two types of assessments may exist. Given that imaging can improve the detection of most recurrences [82], heterogeneity in scanning frequency may account for differences in treatment groups. Therefore, the more frequent scanning intervals in PRIMA [16,40,41,42,43] (performed every 12 weeks) may have led to shorter median PFS estimates compared with PAOLA-1 [19,34] (scans performed every 24 weeks, or every 12 weeks if there was evidence of disease progression) and are therefore a source of bias.

## 4. Discussion

Every day, oncologists must select the best therapeutic option for their patients based on a multitude of clinical trial data, particularly RCTs that are designed to address different unmet needs [1,4]. Clinicians treating women with advanced ovarian cancer face this challenge when selecting among available maintenance therapy options following 1L therapy [2,3]. The PARP inhibitor niraparib has shown clinical benefit as a monotherapy in the PRIMA trial in women with advanced ovarian cancer [16]. In this feasibility study, it was determined that an ITC of the PRIMA data with trials of other 1L maintenance options using an NMA was not possible to conduct based on established guidelines for these types of assessments [83,84]. The PRIMA study population differed markedly from several of the other 1L maintenance studies because it enrolled a high proportion of patients with poor prognostic factors such as VRD following PDS, the attainment of a partial response instead of a complete response to chemotherapy, and the receipt of NACT [16]. Additionally, the study designs, including outcome measurements, for other 1L maintenance RCTs were inconsistent with PRIMA as these studies were designed to test different hypotheses. In PAOLA-1, olaparib plus bevacizumab demonstrated efficacy in the intention-to-treat population in the 1L maintenance setting [19]. The present study also determined that a PAIC using IPD for PRIMA and aggregate data for PAOLA-1 was not feasible given the differences in patient populations, including the treatment that patients received prior to study entry, as well as differences in outcome measurements.

A limitation of this analysis is that the RCTs identified for the NMA were informed by an SLR, with the final list of RCTs influenced by the search strategy, selection criteria, and timing of the review. Additionally, the PRIMA PAIC feasibility assessment was limited to the comparison with PAOLA-1. Feasibility assessments were also largely based on PFS, due to limited common outcomes across RCTs.

Other ITCs of 1L maintenance studies in advanced ovarian cancer have been reported. A PAIC of PAOLA-1 and SOLO-1 study of olaparib monotherapy in women with *BRCA*m ovarian cancer was conducted using IPD from SOLO-1 and the *BRCA*m subset of patients in PAOLA-1 [85]. Data from these studies were pooled and an inverse probability of treatment weights was used to match each arm of PAOLA-1 to the SOLO-1 cohort, such that key baseline clinical and demographic characteristics were similar across populations. This study raised the hypothesis that the combination of olaparib and bevacizumab could provide a potentially meaningful improvement in PFS versus olaparib alone as a maintenance treatment for women with newly diagnosed stage III/IV ovarian cancer with *BRCA*m. Another PAIC compared IPD from a subset of patients in PAOLA-1 (with stage IV disease, stage III with VRD after primary surgery, inoperable stage III disease, or any patient who received NACT) using propensity weights to match the baseline characteristics of the PRIMA population [38]. Both datasets were pooled and treatment efficacy was assessed by weighted Cox regression and Kaplan–Meier methods. The results suggested that adding olaparib to bevacizumab improved PFS in this patient population compared with niraparib or bevacizumab alone. However, several limitations were noted. Their analysis relied on the matching of observed prognostic factors and effect modifiers across the studies to minimize differences in patient characteristics and is therefore subject to assumptions around the absence of unobserved confounders, including differences in geographic locations, the frequency of scan assessments for PFS, and, most importantly, the prior receipt of and responder criteria for bevacizumab treatment alongside chemotherapy in PAOLA-1. Furthermore, reported baseline characteristics for the HR-proficient population in PRIMA were lacking in the reported analysis, meaning formal matching was not possible for this subgroup. Given these limitations, combined with the unanchored nature of the PAIC, these analyses are not a reliable estimation of the relative clinical efficacy of treatment regimens in the PRIMA and PAOLA-1 trials. Considering the significant differences between PRIMA [16,40,41,42,43] and PAOLA-1 [19,34] in terms of trial outcomes, inclusion/exclusion criteria, the use of bevacizumab prior to the study, and the use of NACT, conducting a comparison between the two studies would violate the recommended methodology outlined in the NICE DSU, ISPOR, and Cochrane guidelines, and would not produce reliable results to inform medical decision making [22,23,24,35,86].

## 5. Conclusions

It is important to consider that the total body of evidence informing the utility of a therapy should include both RCTs and ITCs [22]. ITCs are needed to inform comparative efficacy when direct comparisons are unavailable due to differences in trial design, which may be a consequence of a rapidly changing treatment landscape [22]. Based on the evidence presented here, ITCs of 1L ovarian cancer maintenance treatment RCTs are subject to uncontrolled heterogeneity and should not be considered appropriate evidence for use in clinical decision-making or reimbursement decisions. In the absence of ITCs, physicians treating women with ovarian cancer should consider the available RCT data along with individual patient characteristics and management of the toxicity profile of treatment options. Treatment planning and patient education about treatment options should be initiated early in the treatment journey to help oncologists and patients navigate the treatment journey [9,87,88]. Cross-trial comparisons of therapeutic agents in oncology should be made with caution, as the current study demonstrates that several confounding factors can preclude objective systematic comparison between RCTs.

## Figures and Tables

**Table 1:** Sources of heterogeneity that hinder comparability of studies.

Category	Factor
Different quality or methods of randomized trials	Adequate concealment of randomization Blinding Duration of follow-up Lost to follow-up Treatment groups
Confounding factors in relation to participant population	Age Genetic variation Diagnostic workup Intensity of surveillance Stage or duration or disease or condition Severity of disease or condition History of surgery and residual disease Previous therapy
Confounding factors in relation to circumstances	Geography Date of trials
Different treatment	Dose Duration Timing
Different outcome measures and methods of statistical analysis	Definition of outcomes Rating instrument Frequency of measurement Start point of measurement End point of measurement Availability of data

**Table 2:** Reasons for exclusion for each trial from NMA with PRIMA [16,40,41,42,43].

Trial	Study Design Eterogeneity: Lack of Common Comparator within the Network	Patient Population Heterogeneity: Inclusion of Patients with FIGO Stage III Disease with no VRD Following PDS	Outcome Heterogeneity	
			Interim or Immature OS Data	Differing Measurement of PFS and OS Starting Time Point due to Trial Design
SOLO-1 [44,45,46,47,48,49,50,51,52,53,54,55,56]		✓ *	✓	
ICON-7 [68,69]	✓			✓
MIMOSA [57,58]		✓	✓	PFS was not assessed
AGO-OVAR16 [59,60,61,62,63,64,65]		✓		
NCT01227928 [66,67]		✓		
GOG-0218 [70,71]	✓			✓
PAOLA-1 [19,34]	✓	✓	✓	
CHIVA/GINECO [37]	✓	✓	✓	✓
TRINOVA-3 [73]	✓	✓		✓
VELIA/GOG-3005 [74,75]	✓	✓	✓	✓
AGO-OVAR12 [72]	✓			✓

* There was also disparity between *BRCA*m disease biomarker status; only patients with documented *BRCA*m were included. *BRCA*m, breast cancer gene mutation; FIGO, International Federation of Gynecology and Obstetrics; NMA, network meta-analysis; OS, overall survival; PDS, primary debulking surgery; PFS, progression-free survival; VRD, visible residual disease.

## Data Availability

GlaxoSmithKline (GSK) makes available anonymized individual participant data and associated documents from interventional clinical studies that evaluate medicines upon approval of proposals submitted to www.clinicalstudydatarequest.com (accessed on 22 December 2021).

## Supplementary Materials

The following supporting information can be downloaded at: https://www.mdpi.com/article/10.3390/cancers14051285/s1, Table S1: EMBASE, Medline, Medline (R) In-Process search strategy (EMBASE interface) clinical search strategy. Table S2: CENTRAL clinical search strategy. Table S3: Gray literature search strategy.
