# Supplementary material for: Feasibility Study of a Network Meta-Analysis and Unanchored Population-Adjusted Indirect Treatment Comparison of Niraparib, Olaparib, and Bevacizumab as Maintenance Therapies in Patients with Newly Diagnosed Advanced Ovarian Cancer
Source: Cancers (Basel). 2022 Mar 2;14(5):1285. doi: 10.3390/cancers14051285 (PMC8909094; doi:10.3390/cancers14051285)
Supplement: Supplementary file 1 [file cancers-14-01285-s001.zip › cancers-1546758-supplementary.pdf]

Supplementary Materials

Table S1: EMBASE, Medline, Medline (R) In-Process search strategy (EMBASE interface) clinical search strategy.

Index	Description	Search terms	Hits
1	Population	'ovarian cancer'/exp OR 'ovar* cancer*':ti,ab OR 'fallopian tube cancer*' OR 'fallopian tube cancer':ti,ab OR 'peritoneal cancer'/exp OR 'periton* cancer*':ti,ab OR ((ovar* OR fallopian OR 'fallopian tube*' OR periton* OR granulosa OR krukenberg* OR oviduct* OR 'uterine tube*') NEXT/3 (neoplas* OR cancer* OR tumour* OR tumor* OR carcino* OR malignan* OR adenocarcino* OR metast* OR mesothelioma*)) OR dysgerminoma* OR gynandroblastoma*	Original SLR (February 13, 2019):<br>179,027<br><br>Update (27 February 2020):<br>192,065
2	Drug (maintenance)	‘PARP inhibitor’/exp OR ‘PARP inhibitor*’:ti,ab OR ‘poly (ADP-ribose) polymerase inhibitor’:ti,ab OR niraparib/exp OR niraparib:ti,ab OR zejula:ti,ab OR MK4827:ti,ab OR ‘MK 4827’:ti,ab OR olaparib/exp OR olaparib:ti,ab OR lynparza:ti,ab OR ‘AZD 2281’:ti,ab OR AZD2281:ti,ab OR rucaparib/exp OR rucaparib:ti,ab OR veliparib/exp OR veliparib:ti,ab OR talazoparib/exp OR talazoparib:ti,ab OR pazopanib/exp OR pazopanib:ti,ab OR votrient:ti,ab OR bevacuzimab/exp OR bevacuzimab:ti,ab OR avastin:ti,ab OR altuzan:ti,ab	Original SLR:<br>19,930<br><br>Update:<br>23,183
3	Study types:<br>RCT Filter [1]	('clinical trial'/de OR 'randomized controlled trial'/de OR ‘controlled clinical trial’/de OR ‘multicenter study’/de OR ‘Phase 3 clinical trial’/de OR ‘Phase 4 clinical trial’/de OR 'randomization'/de OR 'single blind procedure'/de OR 'double blind procedure'/de OR 'crossover procedure'/de OR 'placebo'/de OR 'randomi*ed controlled trial*':ti,ab OR rct:ti,ab OR 'random allocation':ti,ab OR 'randomly allocated':ti,ab OR 'allocated randomly':ti,ab OR (allocated NEXT/2 random):ti,ab OR 'single blind*':ti,ab OR 'double blind*':ti,ab OR ((treble OR triple) NEXT/1 blind*):ti,ab OR placebo*:ti,ab OR 'prospective study'/de) NOT ('case study'/de OR 'case report':ti,ab OR 'abstract report'/de OR 'letter'/de OR ‘editorial’/de OR ‘note’/de)	Original SLR:<br>1,979,541<br><br>Update:<br>2,156,154

4	Observation study filter [1]	'clinical trial'/de OR 'case control study' OR 'family study'/de OR 'longitudinal study'/de OR 'retrospective study'/de OR ('prospective study'/de NOT 'randomized controlled trial'/de) OR 'cohort analysis'/de OR (cohort NEXT/1 (study OR studies)) OR (('case control' NEXT/1 (study OR studies)):ti,ab) OR (('follow up' NEXT/1 (study OR studies)):ti,ab) OR ((observational NEXT/1 (study OR studies)):ti,ab) OR ((epidemiologic* NEXT/1 (study OR studies)):ti,ab) OR (('cross sectional' NEXT/1 (study OR studies)):ti,ab)	Original SLR: 2,956,349
			Update: 3,284,461
5	Combine filters and restrict date	#1 AND #2 AND (#3 OR #4)	Original SLR: 1,025
		#1 AND #2 AND (#3 OR #4) AND [13-2-2019]/sd NOT [27-2-2020]/sd AND [humans]/lim	Update: 296

RCT, randomized control trial; SLR, systematic literature review. [1] SIGN. Search filter. https://www.sign.ac.uk/what-we-do/methodology/search-filters/ Accessed July 14.

Table S2: CENTRAL clinical search strategy.

Index	Description	Search terms	Hits
1	Terms for population	MeSH descriptor: [Ovarian Neoplasms] explode all trees or MeSH descriptor: [Fallopian Tube Neoplasms] explode all trees or MeSH descriptor: [Peritoneal Neoplasms] explode all trees or 'ovar* cancer':ti,ab or 'fallopian tube cancer':ti,ab or 'periton* cancer*':ti,ab or ((ovar* or fallopian or 'fallopian tube*' or periton* or granulosa or krukenberg* or oviduct* or 'uterine tube*') next/3 (neoplas* or cancer* or tumour* or tumor* or carcino* or malignan* or adenocarcino* or metast* or mesothelioma*)) or dysgerminoma* or gynandroblastoma*	Original SLR: 10,518<br>Update: 13,868
2	Comparator (maintenance)	‘PARP inhibitor*’:ti,ab or ‘poly (ADP-ribose) polymerase inhibitor’:ti,ab or niraparib:ti,ab or zejula:ti,ab or MK4827:ti,ab or ‘MK 4827’:ti,ab or olaparib:ti,ab or lynparza:ti,ab or ‘AZD 2281’:ti,ab or AZD2281:ti,ab or rucaparib:ti,ab or veliparib:ti,ab or talazoparib:ti,ab or pazopanib:ti,ab or votrient:ti,ab or bevacuzimab:ti,ab or avastin:ti,ab or altuzan:ti,ab	Original SLR: 1,073<br>Update: 2,133
3	Combine terms	#1 and #2 in trials.	Original SLR: 260<br>Update: 447
4	Restrict dates	13-2-2019 to 27-02-2020	Update only: 167

MeSH, medical subject heading; PARP, poly (ADP-ribose) polymerase; SLR, systematic literature review.

Table S3: Grey literature search strategy.

Source	Interface/URL	Search strategy and hits
NICE	https://www.nice<br>.org.uk	Original SLR (February 13, 2019):<br>• Ovarian cancer (36) – 0 chosen<br>o Fallopian tube cancer (6) – 0 chosen<br>o Peritoneal cancer (14) – 0 chosen<br>o Olaparib (2) – 0 chosen<br>• Rucaparib (4) – 0 chosen<br>• Bevacizumab (19) – 0 chosen
		Update (February 27, 2020):<br>• Ovarian cancer (19) – 0 chosen<br>o Fallopian tube cancer (10) – 0 chosen<br>o Peritoneal cancer (12) – 0 chosen<br>o Olaparib (6) – 0 chosen<br>o Rucaparib (3) – 0 chosen<br>o Bevacizumab (8) – 0 chosen
PBS	http://www.pbs.<br>gov.au/	Original SLR:<br>• “Ovarian cancer” (36) – 0 chosen<br>• “Fallopian tube cancer” (8) – 0 chosen<br>• “Peritoneal cancer” (28) – 0 chosen
		Update:<br>• “Ovarian cancer” (35) – 0 chosen<br>• “Fallopian tube cancer” (8) – 0 chosen<br>• “Peritoneal cancer” (9) – 0 chosen
CADTH	http://www.cadth.ca	Original SLR:<br>• Ovarian cancer (20) – 0 chosen<br>• Fallopian tube cancer (4) – 0 chosen<br>• Peritoneal cancer (12) – 0 chosen
		Update:<br>• Ovarian cancer (9) – 0 chosen<br>• Fallopian tube cancer (0) – 0 chosen<br>• Peritoneal cancer (5) – 0 chosen
SMC	http://www.scottish<br>medicines.org.uk	Original SLR:<br>• Malignant disease and immunosuppression filter – (74) – 0 chosen
		Update:<br>• Malignant disease and immunosuppression filter – (0) – 0 chosen
ClinicalTrials.gov	https://clinicaltrials.gov<br>/ct2/home	Direct searching of this resource was not necessary as it is catalogued by the Cochrane Registry. (0 chosen)
ESMO	https://www.esmo.org/	Original SLR: (“ovarian” OR “fallopian tube” OR “peritoneal”) AND “cancer” (100) – 0 chosen

		Update: (“ovarian” OR “fallopian tube” OR “peritoneal”) AND “cancer” (40) – 1 chosen
ESGO	https://www.esgo.org/	Original SLR: ESGO 2017 Vienna Congress – 0 chosen
		Update: ESGO 2019 Athens Congress – 2 chosen
ASCO	https://meeting library.asco.org/	Original SLR: (Title:"Ovarian cancer" OR Title:"Fallopian tube cancer" OR Title:"Peritoneal cancer") AND (Title:"cost" OR Title:"economic" OR Title:"utility" OR Title:"utilities" OR Title:"disutilities" OR Title:"eq-5d" OR Title:"resource")) (8) – 1 chosen
		Update: (Title:"Ovarian cancer" OR Title:"Fallopian tube cancer" OR Title:"Peritoneal cancer") AND (Title:"cost" OR Title:"economic" OR Title:"utility" OR Title:"utilities" OR Title:"disutilities" OR Title:"eq-5d" OR Title:"resource") (2) – 1 chosen
ASGO	http://asgo2017 tokyo.umin.jp/ http://www.asgo2019.org /Information/ASGO2019 File/list.asp	Manually search ASGO 2017 and ASGO 2019 – 0 chosen
SGO	http://sgo.peachnewmedia. com/store/provider/custom page.php?pageid=11	The following conferences were manually searched for any relevant information: 2019 SGO Winter Meeting, 2018 SGO Winter Meeting, SGO 2018 Annual Meeting on Women's Cancer, 2018 Allied Health Professionals Workshop: Solutions to Practice Challenges and 2017 Allied Workshop – 0 chosen
NCRI	http://abstracts.ncri.org.uk/	Original SLR:<br>• Ovarian cancer (60) – 0 chosen<br>• Fallopian tube cancer (3) – 0 chosen<br>• Peritoneal cancer (11) – 0 chosen
		Update:

		• Ovarian cancer (19) – 0 chosen<br>• Fallopian tube cancer (0) – 0 chosen<br>• Peritoneal cancer (3) - 0 chosen
EACR	https://www.eacr.org/search/	Original SLR:<br>• Ovarian cancer (8) – 0 chosen<br>• Fallopian tube cancer (0) – 0 chosen<br>• Peritoneal cancer (0) – 0 chosen
		Update:<br>• Ovarian cancer (1) – 0 chosen<br>• Fallopian tube cancer (0) – 0 chosen<br>• Peritoneal cancer (0) – 0 chosen
ISPOR	https://www.ispor.org/heor-resources/presentations-database/search	Original SLR:<br>• Ovarian cancer (68) – 0 chosen<br>• Fallopian tube cancer (3) – 0 chosen<br>• Peritoneal cancer (4) – 0 chosen
		Update:<br>• Ovarian cancer (19) – 0 chosen<br>• Fallopian tube cancer (0) – 0 chosen<br>• Peritoneal cancer (0) – 0 chosen
Google Scholar	https://scholar.google.com/	Original SLR:<br>allintitle: ("ovarian cancer" OR “fallopian tube cancer" OR “peritoneal cancer”) AND ((("trial" OR "study") AND ("randomised" OR "randomized")) OR "rct" OR “observational”) (56) – 5 chosen
		Update:<br>allintitle: ("ovarian cancer" OR “fallopian tube cancer" OR “peritoneal cancer”) AND ((("trial" OR "study") AND ("randomised" OR "randomized")) OR "rct" OR “observational”) (21) – 2 chosen

ASCO, American Society of Clinical Oncology; ASGO, Asian Society of Gynecologic Oncology; CADTH, Canadian Agency for Drugs and Technologies in Health; EACR, European Association for Cancer Research; ESGO, European Society of Gynecological Oncology; ESMO, European Society for Medical Oncology; ISPOR, Professional Society for Health Economics and Outcomes Research; NCRI, National Cancer Research Institute; NICE, National Institute for Health and Care Excellence; PBS, Pharmaceutical Benefits Scheme; RCT, randomized control trial; SGO, Society of Gynecological Oncology; SLR, systematic literature review; SMC, Scottish Medicines Consortium.
